# Targeting Phosphodiesterases—Towards a Tailor-Made Approach in Multiple Sclerosis Treatment

**DOI:** 10.3389/fimmu.2019.01727

**Published:** 2019-07-24

**Authors:** Melissa Schepers, Assia Tiane, Dean Paes, Selien Sanchez, Ben Rombaut, Elisabeth Piccart, Bart P. F. Rutten, Bert Brône, Niels Hellings, Jos Prickaerts, Tim Vanmierlo

**Affiliations:** ^1^Department of Neuroimmunology, European Graduate School of Neuroscience, Biomedical Research Institute, Hasselt University, Hasselt, Belgium; ^2^Department Psychiatry and Neuropsychology, European Graduate School of Neuroscience, School for Mental Health and Neuroscience, Maastricht University, Maastricht, Netherlands; ^3^Department of Morphology, Biomedical Research Institute, Hasselt University, Hasselt, Belgium; ^4^Department of Physiology, Biomedical Research Institute, Hasselt University, Hasselt, Belgium

**Keywords:** multiple sclerosis, phosphodiesterase, neuroinflammation, CNS repair, remyelination

## Abstract

Multiple sclerosis (MS) is a chronic demyelinating disease of the central nervous system (CNS) characterized by heterogeneous clinical symptoms including gradual muscle weakness, fatigue, and cognitive impairment. The disease course of MS can be classified into a relapsing-remitting (RR) phase defined by periods of neurological disabilities, and a progressive phase where neurological decline is persistent. Pathologically, MS is defined by a destructive immunological and neuro-degenerative interplay. Current treatments largely target the inflammatory processes and slow disease progression at best. Therefore, there is an urgent need to develop next-generation therapeutic strategies that target both neuroinflammatory and degenerative processes. It has been shown that elevating second messengers (cAMP and cGMP) is important for controlling inflammatory damage and inducing CNS repair. Phosphodiesterases (PDEs) have been studied extensively in a wide range of disorders as they breakdown these second messengers, rendering them crucial regulators. In this review, we provide an overview of the role of PDE inhibition in limiting pathological inflammation and stimulating regenerative processes in MS.

## Introduction

Multiple sclerosis (MS) is a chronic immune-mediated demyelinating disorder of the central nervous system (CNS) affecting more than 2.5 million people worldwide, making it the most common neurodegenerative disease in young adults (1). Although the exact etiology remains unknown, MS is thought to develop due to an interplay between susceptibility genes and environmental factors that are yet to be fully elucidated (2). The clinical course of MS is characterized by various clinical symptoms, including gradual muscle weakness, fatigue, and cognitive impairment, which arise in either episodic periods or progress during the disease course (3). Current FDA-approved treatments modulate the prominent immune responses of MS, but are unable to halt disease progression (4). Hence, there is an urgent need for the development of new therapeutic strategies. In recent decades, phosphodiesterase (PDE) inhibitors have shown to exhibit immunomodulatory and neuroprotective functions rendering them interesting candidates for the management of MS disease.

Clinically, MS can be divided in three distinct classifications: relapsing remitting MS (RRMS), primary progressive MS (PPMS) and secondary progressive MS (SPMS). RRMS is the most frequent subtype, affecting approximately 85% of MS patients and can be recognized by periods of remittance (5–7). This early stage of MS is characterized by the presence of active, inflammatory lesions characterized by perivenular infiltration of myelin-reactive lymphocytes and macrophages, resulting in demyelination of the axonal branches (5–7). These inflammatory relapses are followed by the activation of an endogenous repair mechanism called remyelination, resulting in a period of functional recovery (5–7). Fifty percent of RRMS patients undergo a transition to the progressive form of the disease within a period of 15 years, labeled SPMS (8, 9). Additionally, ~15% of MS patients are classified as PPMS and endure gradual accumulation of disability from disease onset without experiencing an initial relapsing course (10). Despite a decrease in frequency of new lesion activity during these chronic stages, there is an accumulation of chronically demyelinated lesions accompanied by an increase in neurological deficits, and a gradual decline in motoric and cognitive function (9). These chronically demyelinated lesions are characterized by a reduced number of oligodendrocytes, as well as the formation of astrogliotic scar tissue and prominently demyelinated axons, subsequently leaving axons vulnerable to axonal transection (11).

The pathogenesis of MS is thought to be driven by the massive extravasation of myelin-reactive T and B lymphocytes into the CNS across the blood-brain barrier (BBB) (12). Perivenular infiltration of these auto-reactive lymphocytes disturb the homeostatic immune balance in the brain, leading to a pro-inflammatory microenvironment and subsequent CNS damage (13). Despite this, phagocytes are the principle effector cells during the neuroinflammatory and neurodegenerative processes of MS and include infiltrated monocyte-derived macrophages and brain resident microglia and macrophages (14). In MS, the disturbed homeostatic balance in the CNS skews the activation status of macrophages and microglia, subsequently fueling the neuroinflammatory response, or ceasing the inflammatory process through exerting neuroprotective functions (15). However, in the early course of MS, neuroinflammation not only induces demyelination but, it also activates remyelination. Early remyelination in active MS lesions is characterized by the expansion and mobilization of oligodendrocyte precursor cells (OPCs) (5–7, 16–19). Despite the presence of sufficient numbers of OPCs in the vicinity of pathological lesions, endogenous repair mechanisms gradually fail when disease progresses, resulting in chronically demyelinated axons embedded in gliotic scar tissue (20–24). When remyelination is not initiated, loss of myelin disrupts axonal function in addition to compromising the physical integrity of axons by increasing susceptibility to inflammatory mediators, glutamate mediated toxicity, and the disrupted trophic support provided by myelinating oligodendrocytes (25). It follows that, axonal ovoids, a hallmark of transected axons, are profoundly present in MS tissue (26).

**Figure 1:**
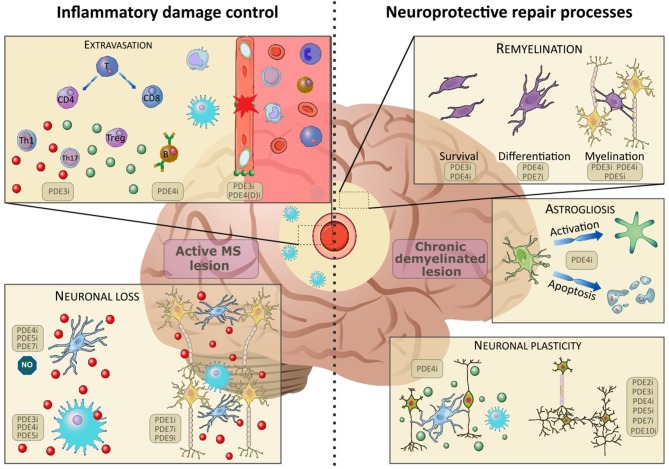
The effects of PDE inhibition on different cell types in inflammatory damage control (active lesion) and neuroprotective repair processes (chronic demyelinated lesion). Upon disruption of the blood-brain-barrier (BBB), patrolling immune cells (monocytes, T- and B-lymphocytes) extravasate into the central nervous system. Here, the immature cells differentiate and elicit their functions in the inflammatory environment. A broad spectrum of pro- (red) and anti-(green) inflammatory cytokines are found in the active lesion. Inhibition of PDEs has been found to positively influence BBB integrity, B-cell functioning and T-cell expression patterns, skewing them toward an anti-inflammatory phenotype. The inflammatory environment causes activation of microglia and infiltrating macrophages, which contributes to excessive neuronal loss. Inhibition of PDEs counteracts this inflammatory activation and promotes neuronal survival. In chronic demyelinated lesions, the inhibition of PDEs has been found to ameliorate remyelination, thus supporting endogenous repair mechanisms. Furthermore, PDE inhibition counteracts astrogliosis by halting activation and apoptosis of astrocytes. Finally, inhibition of a multitude of PDEs has been found to promote neuronal plasticity and skew microglia and infiltrating macrophages toward an anti-inflammatory phenotype. Images were modified from Reactome icon library and Servier Medical Art, licensed under a Creative Common Attribution 3.0 Generic License (42).

Interestingly, cyclic nucleotide signaling pathways, such as cyclic 3′-5′ adenosinemonophosphate (cAMP) and cyclic 3′-5′ guanosinemonophosphate (cGMP) have been shown to be responsible for a variety of intracellular processes involved in both neuroinflammation and CNS repair processes (27–31). Therefore, orchestrating cellular responses by altering the intracellular balance of cyclic nucleotides can be considered an important therapeutic strategy to modulate the pathogenesis of MS (27, 32). Upon an extracellular trigger, cyclic nucleotides are formed as second messengers to amplify the incoming signal, subsequently activating protein kinases, and ion channels. Cyclic nucleotides orchestrate divergent key cellular processes such as cellular differentiation and maturation (33). cAMP and cGMP are synthesized by adenylyl cyclase (AC) and guanylyl cyclase (GC), respectively. AC converts adenosine 5′-triphosphate (ATP) into cAMP while guanosine 5′ triphosphate (GTP) is the substrate for GC to synthesize cGMP. In contrast, intracellular cyclic nucleotide levels are spatiotemporally regulated by the presence of PDEs (27). PDEs comprise a superfamily of enzymes that catalyze the hydrolysis of intracellular cyclic nucleotides. PDEs can be categorized into eleven PDE families (e.g., PDE1-11) that jointly cover 21 PDE genes (e.g., PDE4A-PDE4D) (33–35). Interestingly, each of these genes codes for different isoforms (e.g., PDE4B1-5), yielding a total of at least 77 different protein-coding isoforms. PDE gene families, genes, and isoforms can be distinguished based on their subcellular distribution, enzymatic activity, kinetic properties, and substrate specificity (36, 37). Five PDE families have a dual substrate specificity, meaning they can hydrolyze and inactivate both cAMP and cGMP (PDE 1, 2, 3, 10, and 11) (34). The remaining six PDE families specifically and exclusively hydrolyze cAMP (PDE 4, 7, and 8) or cGMP (PDE 5, 6, and 9). The cell type-specific PDE expression of the isoforms yields a specific fingerprint that provides an incentive to develop custom-made PDE-targeting strategies (35, 38, 39). Different small molecules directed against specific PDE families, genes, or isoforms have been tested in the context of neurodegeneration, neuroinflammation, and CNS repair (29–31, 40, 41). In this review, we discuss experimental studies and clinical implications regarding PDE inhibition as a strategy for inflammatory damage control and stimulation of related repair processes in MS (Figure 1).

## Inflammatory Damage Control in Active MS Lesions by Inhibiting PDEs

Ceasing the inflammation that drives the neuroinflammatory and neurodegenerative responses in MS is considered a valuable therapeutic strategy. PDEs have been extensively studied for their anti-inflammatory properties. Several processes can be targeted to diminish the inflammatory response. PDE inhibitors are of interest due to their potential to (1) strengthen the BBB to prevent peripheral lymphocyte accumulation in the CNS, (2) restore the balance between pro-inflammatory and anti-inflammatory mediators including lymphocytes and phagocytes, and (3) prevent astrogliotic scar formation. Each of these potential aspects is further detailed below.

### Blood-Brain Barrier

The BBB is comprised of smooth muscle cells, endothelial cells, pericytes, and astrocytic endfeet, functioning as a barrier to restrict the entrance of peripheral immune cells and toxic molecules into the CNS (12). In early MS development, pro-inflammatory lymphocytes activate the endothelial cells of the BBB. Endothelial activation leads to an upregulation of cell adhesion molecules that promote the massive infiltration of myelin-reactive lymphocytes into the CNS (13, 43). Endothelial cells are linked by multiprotein complexes called tight junctions, which become dysfunctional in early MS development. Therefore, restoring the loosened tight junctions, and stabilizing the BBB can prevent further infiltration of immune cells into the CNS, subsequently halting or reducing disease progression.

The involvement of cAMP in endothelial barrier functions has been extensively studied. cAMP analogs, such as dibutyryl cAMP (dbcAMP), can decrease junctional permeability and therefore diminish trans-endothelial transport of both small and large molecules (44). Nevertheless, it is compartmentalized cAMP generation rather than the general accumulation of intracellular cAMP that coordinates barrier preservation or destabilization (45). Vascular permeability is enhanced when cytosolic cAMP is increased, while barrier integrity is maintained when cAMP accumulates in cellular vacuoles (45). In contrast to cAMP, the outcome of directly increasing cGMP levels on endothelial barrier function is yet to be elucidated. However, indirectly raising intracellular cGMP levels, by increasing NO signaling has been shown to induce vascular smooth muscle relaxation, increase BBB permeability, and inhibit endothelial cell apoptosis (46, 47). Based on these results, increasing cGMP signaling does not seem to be a suitable therapeutic strategy for restoring BBB integrity during inflammatory relapses in MS. Therefore, solely cAMP or dual-substrate PDE inhibitors are discussed here as a therapeutic strategy for reducing BBB disruption.

In particular, the cAMP-specific PDE4 inhibitors have been evaluated for their potential to strengthen the BBB. In experimental autoimmune encephalomyelitis (EAE), a neuroinflammatory animal model for MS, the pan PDE4 inhibitor rolipram (2 mg/kg, i.p injected twice a day) modified the cerebrovascular endothelial permeability and thereby restored BBB function (48). The same protective features of rolipram were observed in an animal model for stroke, where treatment preserved the expression of the tight junction proteins occludin and claudin-5 (49). Furthermore, the inhibition of PDE4D is of particular interest for altering BBB permeability since it co-localizes with the endothelial marker RECA-1 and the vascular smooth muscle cells α-SMA (50). However, the exact role of PDE4D in restoring BBB integrity remains to be elucidated. Both cAMP-specific PDE inhibitors and dual-substrate PDE inhibitors have been proposed as potential therapeutic targets. As such, administration of the PDE3 inhibitor cilostazol to a murine model for age-related cognitive impairment (1.5% w/w) over a 3 months period increased the amount of zona occludens protein 1 (ZO-1) and occluding, subsequently improving BBB integrity (51). Therefore, PDE inhibitors acting on the cAMP pathway are predicted to strengthen BBB functionality. Due to the opposing outcomes upon elevating cAMP in different subcellular compartments, elucidating which PDE enzymes are present in endothelial vacuoles and absent in the cytosol can hold the key for identifying which PDE needs to be targeted for restoring BBB integrity. Unraveling essential signaling peptides during translation will become indispensable for determining PDE compartmentalization.

### Lymphocytes

Disrupted BBB integrity in MS patients facilitates peripheral immune cell infiltration. The two main subsets of infiltrating T lymphocytes in MS are CD^4+^ and CD^8+^ T cells (52). Various subsets of CD^4+^ T helper cells have been identified based on their cytokine secretion profiles. In particular, CNS Th1 and Th17 cell frequencies are increased in RRMS patients compared to healthy controls. Cytokines secreted by the different T cell subsets are critical mediators of the neuroinflammatory response. Upon activation, Th1 cells aggravate the neuroinflammatory response by secreting pro-inflammatory cytokines [e.g., tumor necrosis factor α (TNFα), interleukin 1β (IL-1β), and interferon-γ (IFN-γ)], subsequently promoting cellular infiltration and activation of phagocytes and B cells (53). Th17 cells are mainly characterized by their production of interleukin 17 (IL-17), but exert a polyfunctional phenotype depending on their overall cytokine secretion profile. Pathogenic Th17 cells aggravate inflammatory processes by producing high levels of the pro-inflammatory cytokine IFN-γ, whereas non-pathogenic Th17 cells produce more protective cytokines such as IL-10 (54). IL-17 levels are elevated in the serum and cerebrospinal fluid (CSF) of MS patients and are correlated with MS disease severity, consequently suggesting a pathogenic role of Th17 cells in MS (55, 56). Moreover, regulatory CD^4+^ T cells (Tregs) from the peripheral blood of RRMS patients show a reduced suppressive capacity, suggesting Treg dysfunction in early MS stages (57). Treg formation is the result of activation of the dominant transcription factor forkhead box P3 (Foxp3) and by producing immunosuppressive cytokines [e.g., transforming growth factor β (TGF-β) and IL-10], Tregs inhibit auto-aggressive T cell responses (58). In addition to CD^4+^ T cells, autoreactive cytotoxic T cells (CD^8+^ T cells) are actively involved in MS pathogenesis. CD^8+^ T cells are found in large numbers in MS lesions in close proximity to damaged oligodendrocytes (59, 60). Therefore, halting MS disease progression can be accomplished by modulating lymphocyte responses through the restoration of second messenger levels using PDE inhibitors.

In the context of T lymphocyte proliferation, differentiation and activation, cAMP is the most extensively studied second messenger. Increasing cAMP levels attenuates the T lymphocyte-mediated immune response by reducing the production of pro-inflammatory cytokines (e.g., IFN-γ, TNF-α, and IL-1β), T cell proliferation and T cell activation (61–63). Increased levels of cAMP further drive the development of Tregs to maintain immunological homeostasis by suppressing the innate immune responses (63). Recently, it has been reported that anti-CD3/CD28 stimulation to activate naïve CD^4+^ T cells increased the enzymatic levels of PDE7, particularly the expression of the PDE7A1 isoform (64, 65). Accordingly, in EAE mice where T cells are highly activated, the PDE7 inhibitor TC3.6 was shown to increase mRNA levels of FoxP3 and augment the production of IL-10. Additionally, PDE7 inhibition was accompanied by decreased levels of IL-17 and reduced T cell proliferation (66). Conversely, PDE7A knockout mice did not show a difference in T cell activation and cytokine production, obfuscating the role of PDE7 in T cell-mediated immune responses and raising the possibility of an indirect effect of the PDE7 inhibitor TC3.6 (67). PDE4 is the most extensively studied cAMP-specific PDE in the context of modulating pro-inflammatory processes. As observed with TC3.6, inhibiting PDE4 decreased T cell proliferation and reduced the production of pro-inflammatory cytokines (TNF-α and IL-17) while increasing the production of anti-inflammatory cytokines (IL-10) in EAE mice (29, 66). Symptomatic treatment with 2.5 mg/kg of the PDE4 inhibitor rolipram decreased the number of perivascular inflammatory infiltrates and was accompanied by a reduction of clinical symptoms in EAE mice (29, 66). Interestingly, upon anti CD3/CD28 co-stimulation of either human CD4+ naive or memory T cells, the enzymatic activities of PDE4A and PDE4D alone were upregulated, although mRNA levels of PDE4A, PDE4B, and PDE4D were increased (68). Furthermore, knockdown of PDE4D in these activated human CD4+ T cells, using siRNA reduced their proliferation rate and inhibited the secretion of IFN-γ (68). In EAE mice, mRNA levels of the PDE4B2 isoform were increased in infiltrating T cells in the CNS (30). This increase in PDE4B2 was positively correlated with FoxP3 and TGF-β mRNA levels, suggesting a modulatory role for PDE4B2 in Treg regulation (30, 66, 69). Based on these findings, cAMP-specific PDE inhibition in T cells can lower the inflammatory cytokine production by acting directly on Th1 and Th17 cells or by regulating the immune response through Treg cells. Furthermore, the dual substrate PDE3 inhibitor cilostazol has been shown to ameliorate encephalitogenic specific T cell responses in EAE mice by reducing lymphocytic proliferation and IFN-γ production in the CNS (70). These findings are consistent with the previous observations using cAMP-specific PDE inhibitors. Despite this, an involvement of cGMP in T cell regulation cannot be excluded as cGMP has been shown to be highly expressed in the cytoplasm of T cells (71–73). Upon NO treatment, T cell adhesion to ICAM-1 and PECAM-1 on endothelial cells of the human brain microvasculature is reduced in a cGMP-dependent manner (74). Accordingly, increasing both cAMP and cGMP by inhibiting specific PDEs can be considered as a potential therapeutic strategy to limit T cell activation by either lowering the pro-inflammatory cytokine production by Th1 and Th17 cells, or by enhancing the suppressive capacity of Tregs.

Although the perivenular infiltration of B cells is less prominent compared to T cells in MS, their contribution to the pathogenesis is highlighted by the anti-CD20 monoclonal antibody therapy that induces B cell depletion and subsequently limited the number of relapses in RRMS patients (75, 76). B cells exert a central role in the pathogenesis of MS by their antibody-independent functions that can either activate or suppress inflammatory responses (77). However, not much is known about second messenger signaling in B lymphocytes. Opposing results were reported depending on the nature of second messengers in B cell cycling (78). Treating murine B lymphocytes with the neurotransmitter acetylcholine indirectly increased the intracellular cGMP levels by stimulating the NO/cGMP pathway, consequently stimulating B cells to enter the cell cycling stages (78, 79). In contrast, adrenaline-induced intracellular cAMP inhibited the entry of B lymphocytes into the DNA replication stage of the cell cycle (78). The latter is consistent with the observations that forskolin, an activator of AC in the plasma membrane, arrested human B lymphocytes in the G1 phase of cell cycling and thereby inhibited B cell growth (80). Furthermore, forskolin promoted apoptosis in human resting B cells (80). However, there is little evidence that PDE inhibitors are able to modulate B cell responses. The PDE4 inhibitors apremilast, rolipram and Ro 20-1724 did not affected B cell differentiation, however they did inhibit IgE production in human PBMCs after IL-4 stimulation (81, 82). This decrease in IgE production was not observed upon PDE3 or PDE5 inhibition, which can be explained by the marginal PDE3 activity and lack of PDE5 activity in healthy B lymphocytes (81, 83, 84). While there is currently little evidence for a direct effect, indirect effects of PDE inhibitors on B cell responses in the pathogenesis of MS cannot be ruled out.

### Phagocytes

Another strategy to control the inflammatory process in MS involves modulating the response of phagocytes in the CNS. In the CNS, phagocytes actively survey the CNS microenvironment in search of harmful pathogens and damage signals (85). In order to retain CNS homeostasis, phagocytes orchestrate different processes including synaptic pruning, shaping neurogenesis, and clearance of cellular debris and apoptotic neurons (86, 87). Depending on the environmental stimuli, phagocytes cover a divergent spectrum of activation states. Upon classical activation (e.g., IFN-γ as activation stimulus), macrophages and microglia polarize toward a more pro-inflammatory phenotype. These classically activated phagocytes contribute to the inflammatory response by producing pro-inflammatory cytokines and chemokines (e.g., TNFα and IL-1β) and therefore mediate tissue damage (15). In contrast, upon alternative activation (e.g., IL-4 as activation stimulus), phagocytes polarize toward a more anti-inflammatory phenotype. These alternatively activated phagocytes are characterized by the production of anti-inflammatory cytokines (e.g., TGFβ and IL-10) and growth factors (e.g., IGF and BDNF). Additionally, anti-inflammatory phagocytes facilitate the clearance of cellular debris which enables the initiation of repair processes (15). It is postulated that persistent neuroinflammatory processes create an imbalance between pro-and anti-inflammatory phagocytes, resulting in neurotoxicity and subsequent neurodegeneration (88).

Interestingly, murine studies using EAE have demonstrated that a phenotypic switch of phagocytes from the pro- to the anti-inflammatory phenotype is associated with milder clinical scores (89). Moreover, after focal LPC-induced demyelination, pro-inflammatory phagocytes seem to drive OPC proliferation. However, it is the later switch to the pro-reparative phenotype that is necessary for OPC differentiation into mature myelinating oligodendrocytes that establish functional remyelination (89). Additionally, anti-inflammatory phagocytes are critical contributors in ceasing the inflammatory response and allowing CNS repair (89). Balancing the levels of cAMP and cGMP in phagocytes is considered critical for orchestrating phagocyte polarization and organizing phagocytosis (90). However, abnormally high levels of cAMP inhibit myelin phagocytosis *in vitro*, even though increasing cAMP skews the polarization toward an anti-inflammatory phenotype characterized by high levels of arginase 1 (Arg1) (90, 91). Furthermore, cGMP has been reported to be associated with the actin cytoskeleton in phagocytes (64, 65, 92, 93). Stimulating the cGMP-PKG pathway dramatically reorganized the actin cytoskeleton of microglia, giving them a phagocytosis-promoting morphology and subsequently enhanced clearance of apoptotic cells and cell debris (94, 95). In MS, internalization of myelin debris by phagocytes at the lesion site is crucial for allowing endogenous remyelination. Therefore, increasing intracellular cAMP or cGMP levels in phagocytes can either alter the inflammatory responses in the CNS or promote clearance of debris, respectively, and can be considered a potential therapeutic strategy for MS.

In murine monocytes and macrophages, the PDE4B gene in particular has been related to inflammatory responses (96). Accordingly, PDE4B inhibition enhanced the secretion of the anti-inflammatory IL-1 receptor antagonist (IL-1Ra) in PDE4B^−/−^ macrophages, at least partially through promoting the phosphorylation and subsequent activation of signal transducer and activator of transcription 3 (STAT3) (97). Furthermore, a positive correlation between PDE4B2 in APC cells (e.g., microglia and macrophages) and the clinical scores of EAE mice was observed (30). Transcriptional upregulation of PDE4B2 is predicted to mediate the activation of the toll receptor-4 pathway, characterized by the production of the pro-inflammatory cytokine TNF-α (98–101). Multiple studies suggest that peripheral inflammation is linked to the development of neuroinflammation (102, 103). In both humans and mice, spinal cord injury (SCI) triggered the expansion of the proteobacteria phylum, leading to an increased systemic endotoxemia that allows LPS from intestinal bacteria to enter the bloodstream, subsequently activating peripheral monocytes and macrophages (103–105). Subsequently, when inducing SCI in PDE4B knockout mice, inflammatory responses, and endoplasmic reticulum (ER) stress were significantly decreased within the spinal cord (SC) of these mice, suggesting the critical involvement of PDE4B in (neuro) inflammatory responses potentially occurs by suppressing monocyte and macrophage activation (103). As with SCI, alcohol consumption induces endotoxemia and subsequent peripheral monocyte activation (106). In mice, alcohol-induced endotoxemia induced PDE4B expression in both peripheral monocytes and CNS resident microglia. This induced PDE4B expression was characterized by a decrease in cAMP levels and subsequent glial activation, indicating a potential pathogenic role of Pde4b in alcohol-induced neuroinflammation (106). Besides PDE4, PDE5 inhibitors sildenafil, and vardenfil have been studied for their effects on macrophage phenotype and CNS infiltration. Sildenafil treatment (10 mg/kg, daily s.c injected) improved clinical scores in EAE mice and increased the expression of Ym-1, a canonical anti-inflammatory macrophage marker in the SC of these mice. In addition, PDE5 inhibition promoted phagocytosis of myelin debris (89). Therefore, by inhibiting cAMP-specific PDEs, this pro-inflammatory response can be diminished and disease progression can be halted. In contrast, inhibition of cGMP-specific PDEs does not actively suppress the pro-inflammatory responses of infiltrating macrophages, but rather increases the phagocytosis rate, thereby promoting CNS repair processes.

Moreover, the role of PDEs in macrophage responses has been studied independently of pathological MS processes. For example, PDE3B has been implicated in regulating inflammasome activation of infiltrating macrophages in white adipose tissue (WAT). As such, PDE3B knockout mice displayed reduced serum levels of pro-inflammatory cytokines such as IL1β and TNFα in a peripheral lipopolysaccharide (LPS) challenge. PDE3B ablation significantly reduced macrophage infiltration in WAT of high fat diet-induced obesity mice (107). Additionally, PDE4 inhibition has been shown to reduce clinical symptoms of inflammatory diseases including arthritis and psoriasis by shifting the phenotypic balance of phagocytes (108–110). PDE4 inhibition with apremilast reduced dermal fibrosis by interfering with the release of IL-6 by anti-inflammatory macrophages. This resulted in a decreased fibroblast activation and collagen release in a skin fibrosis mouse model (111). The beneficial effects of PDE inhibition by macrophages that infiltrate the peripheral tissues give rise to multiple implications that are potentially exploitable in those that infiltrate the CNS. Understanding the role of PDE3B ablation as well as the inhibition of PDE4 and PDE5 in promoting macrophage phenotypic shifts in other pathological contexts can be implicated for controlling the phagocyte-related inflammatory responses in MS pathogenesis.

In microglia, PDE4 is the predominant negative regulator of cAMP (112). Roflumilast-mediated PDE4 inhibition increased the mRNA and protein levels of Arg1, skewing polarization of an anti-inflammatory phenotype in myelin-laden microglia, subsequently promoting repair processes in aged rats subjected to chronic cerebral hypoperfusion (113). Inhibiting PDE4 suppresses LPS-mediated release of TNF-α and NO by activated microglia (99). However, this reduction in NO production is abolished when co-culturing microglia with neurons, casting doubt upon this mechanism *in vivo* (99). Furthermore, the novel PDE4 inhibitor FCPR03 suppressed the release of pro-inflammatory cytokines (TNF-α, IL-1β, and IL-6) *in vitro* in LPS stimulated BV2 microglia, and *in vivo* in the hippocampi and cortices of mice peripherally treated with an LPS bolus (114). Interestingly, inhibition of neuroinflammation was abolished when BV2 microglia were pretreated with a PKA inhibitor H89 (114), indicating that the cAMP-downstream PKA/CREB signaling pathway may be responsible for the suppressed production of pro-inflammatory cytokines upon FCPR03 treatment (114). Moreover, the cAMP/PKA signaling pathway inhibits NF-κB, thereby further suppressing neuroinflammation (114). The novel PDE4 inhibitor roflupram enhanced autophagy in both BV2 microglia and in microglia of mice peripherally injected with LPS (115). Autophagy is a process critically involved in maintaining homeostasis as it modulates inflammasome activation and IL-1β production by removing damaged mitochondria (116). Damaged mitochondria are an important source of ROS production that subsequently activates NLRP3-mediated inflammasome activation and IL-1β production (116). By inducing autophagy, roflupram suppressed inflammasome activation, and IL-1β, consequently reducing neuroinflammatory responses in LPS challenged mice (115). Similar effects on autophagy and inflammasome activation were observed when PDE4B was specifically knocked down in primary microglia cells (115). The likely involvement of PDE4B in suppressing inflammatory responses is reinforced by the observation that ABI-4, a PDE4D-sparing PDE4 inhibitor, has been shown to reduce the release of TNF-α in LPS stimulated primary murine microglia (117). Likewise, pre-symptomatic treatment with the PDE7 inhibitor TC3.6 reduced microglial activation in an animal model for PPMS by decreasing a wide range of mediators of the neuroinflammatory processes including IL-1β, TNF-α, IFN-γ, and IL-6 in the SC (118). Furthermore, increasing intracellular cGMP levels by inhibiting PDE5 after LPS stimulation decreased microglial NO, IL-1β and TNF-α production (119). In line with this, the PDE5 inhibitor sildenafil alleviates hippocampal neuroinflammation by normalizing microglial morphology and reducing microglial activation as shown by a diminished IL-1β production (120–122). Moreover, the highly selective PDE10A inhibitor TP-10 reduced the number of CD11b^+^ reactive microglial cells in the striatum and thereby ameliorated brain pathology in an animal model for Huntington's disease, demonstrating its therapeutic potential in MS pathology (123). Furthermore, 10 μM ibudilast, a non-selective PDE inhibitor targeting multiple PDE families (e.g., PDE4 and PDE10), suppressed TNF-α production in activated microglia but lacked efficacy in lowering other pro-inflammatory mediators such as IL-1β or IL-6 (124). Therefore, inhibiting PDEs independently of their substrate specificity in microglia diminishes pro-inflammatory responses and microglia reactivity.

Taken together, different PDE inhibitors can be considered a powerful therapeutic option for ceasing the inflammatory response in MS by altering the balance between cytotoxic and reparative phagocytes. Particularly PDE4B was shown to be critically involved in neuroinflammatory responses, making it an interesting target for developing MS therapies. Furthermore, PDE4B is upregulated in phagocytes following peripheral inflammation, and subsequently aggravates neuroinflammatory responses; a key process in the pathogenesis of MS. Therefore, interfering with this peripheral-central immunological cross-talk by inhibiting specifically PDE4B is an interesting strategy for treating RRMS patients that needs to be explored further.

### Astrocytes

Astrocytes are the most abundant cells of the CNS and exert pleiotropic functions to protect and support other CNS cell types (125, 126). Due to their ideal position in the brain microvasculature, astrocytes can directly respond to infiltrating immune cells during the initial processes occurring in MS (127). Astrocytes produce growth factors and metabolites in order to maintain the homeostatic balance in the brain, but also ensure synaptic and BBB integrity (125, 126). However, during profound CNS injury, astrocytes become highly activated and undergo morphological and functional changes, yielding astrogliosis (128).

In an attempt to investigate whether PDEs are implicated in astrogliosis, it was shown that TLR signaling induced an upregulation of PDE4B, and more specifically increased the protein level of the PDE4B2 isoform (129). Accordingly, twice daily administration of ibudilast (20 mg/kg), a non-specific PDE inhibitor with preferential affinity for PDE4, reduced astroglial activation in an animal model for Parkinson's disease (130). The same results were observed in a rat model for ocular hypertension, in which a decrease in gliosis was accompanied by decreased levels of pro-inflammatory mediators and enhanced neuroviability (131). Interestingly, ibudilast treatment was also demonstrated to prevent astrocyte apoptosis by increasing cGMP levels, suggesting a potential protective role for cGMP-specific PDE inhibitors (132). In line with this hypothesis, PDE5 inhibition by administering 10 μM sildenafil was shown to restore LPS-induced inflammation in astrocytes *in vitro*, as demonstrated by de Santana Nunes et al. (133). In relation to BBB disruption and immune cell infiltration, it is known that astrocytes express lymphocyte adhesion molecules such as ICAM-1 and VCAM-1 in inflammatory states (134). Elevation of intracellular cAMP levels counteracts the inflammatory activation of astrocytes, resulting in a downregulation of these adhesion molecules (135). As such, astrocytic cAMP signaling plays a prominent role in the prevention of peripheral lymphocyte infiltration. Aforementioned studies show that PDEs are greatly involved in the inflammatory aspect of astrocyte biology and that inhibition of selected PDE isoforms can result in the attenuation of astrogliosis.

## Inhibiting PDEs to Boost Repair in Chronically Demyelinated MS lesions

Neuroinflammation and axonal demyelination associated with MS render neurons more vulnerable to degeneration. Stimulating repair in chronically demyelinated MS lesions is a promising strategy for treating progressive MS patients. The main processes to be addressed for boosting this repair include the stimulation of OPC differentiation into myelinating oligodendrocytes, remodeling of the existing neuronal circuits by enhancing neuro-plasticity/-protection to strengthen axonal conduction, and resolving inflammation that allows for phagocytic growth factor secretion (discussed above).

### Oligodendrocytes

In the CNS, the myelinating cells responsible for remyelination are oligodendrocytes which function to maintain neuronal integrity, and facilitate signal conduction in the brain and spinal cord (136, 137). However, oligodendrocytes are known to be extremely vulnerable to damaging signals, such as neuroinflammatory attacks, or ischemic episodes (138, 139). Loss of oligodendrocytes can result in axonal damage and ultimately leads to demyelination and subsequent neurodegeneration. In an attempt to restore this loss of oligodendrocytes, newly myelinating cells can be formed by differentiation of OPCs into mature myelinating oligodendrocytes (137).

cAMP is a key driver of OPC differentiation (140). *In vitro* treatment of OPCs with cAMP analogs, such as dbcAMP or 8-bromo cAMP, support OPC differentiation based on the number of myelin basic protein (MBP) positive cells (141). A similar level of differentiation is observed when using forskolin (142). Accordingly, cAMP-specific PDE inhibitors are thought to stimulate oligodendrocyte development. Treatment of human OPCs with the PDE7 inhibitors TC3.6 or VP1.15 promotes their survival and accelerates their differentiation into mature myelinating oligodendrocytes by stimulating the ERK signaling pathway (143). In parallel, the potent PDE4-inhibitor rolipram (0.5 μM) was shown to boost rat OPC differentiation *in vitro* by increasing the percentage of MBP^+^ cells (140). Furthermore, based on G-ratio analysis, rolipram (0.5 mg/kg/day) enhanced remyelination in the caudal cerebellar peduncle following focal ethidium bromide-induced demyelination *in vivo* (140). In the presence of myelin-associated inhibitors, OPC differentiation is impaired *in vitro* due to an impairment in Erk1/2, p38MapK and Creb1 phosphorylation. However, 0.5 μM of rolipram treatment overcame the inhibitory effects of myelin protein extracts *in vitro* and relieved the induced differentiation block (140). Interestingly, daily administration of 0.5 mg/kg rolipram (by means of s.c. placed minipump) also appeared to protect oligodendrocytes from secondary cell death following experimental SCI, thereby highlighting its multifaceted mode of action during neurodegeneration (144). In relation to oligodendroglial cell death following ischemia, Nobukazu Miyamoto et al. administered 0.1% of a PDE3 inhibitor mixed in regular chow diet up to 28 days to rats suffering from chronic cerebral hypoperfusion. At a cellular level, this resulted in a strong increase of newly generated oligodendrocytes and a subsequent enhanced rate of remyelination in hypoperfusion-induced white matter lesions after bilateral common carotid artery ligation. Even though PDE3 is classified as a dual cAMP/cGMP hydrolyzing enzyme, Miyamoto and colleagues solely investigated the PDE3 inhibition-mediated increase of cAMP and therefore attributed the positive effects on ischemic white matter injury mostly to a cAMP/PKA-mediated pathway (145). Nevertheless, a role for cGMP involvement cannot be excluded in oligodendrocyte differentiation processes. In particular, an increase in nitric oxide (NO)-induced cGMP signaling has been shown to be directly related to oligodendrocyte maturation as determined by an increased MBP and MOG protein expression level (146). The observed increase of maturation supports the rationale that cGMP-specific PDE inhibitors can also exert a positive effect on oligodendrocyte-mediated repair mechanisms. Accordingly, treatment of organotypic cerebellar brain slices with the widely known PDE5 inhibitor, sildenafil (1 μM) for 10 days enhanced the level of remyelination (89). Additionally, in the SC of EAE mice, treated with 10 mg/kg sildenafil once a day for 15 consecutive days (subcutaneous injection), oligodendrocyte maturation was induced in a cGMP-NO-protein kinase G (PKG)-dependent manner (89). Furthermore, sildenafil appeared to have a protective effect in a mouse model for demyelination, as demonstrated by a preserved myelin, and axonal ultrastructure (147). Yet, these protective features of sildenafil are inconsistent with the findings of Muñoz-Esquivel and colleagues. Here, it was reported that sildenafil treatment diminished myelin expression and increased the expression of negative regulators of myelin (Id2 and Id4), which was consistent with the decreased myelination capacity of sildenafil treated oligodendrocytes (148).

Opposing results regarding the *in vitro* effects of sildenafil on OPC differentiation can be potentially attributed to the difference in inhibitor concentrations. In a later study conducted by Muñoz-Esquivel and colleagues, an inhibition in myelin protein expression was observed after 7 days of 50 μM sildenafil treatment. The myelination-promoting effects of sildenafil in organotypic cerebellar brain slices were observed after 1 μM treatment for 10 days. Furthermore, the diminished expression of myelin proteins after sildenafil treatment was observed in pure primary rat OPC cultures, while organotypic cerebellar brain slices contain multiple cell types. Therefore, an indirect effect of sildenafil for promoting remyelination cannot be ruled out. This difference in treatment regimens is a potential explanation for the observed differences and underscore the importance of cGMP fine-tuning. Altogether, both cAMP and cGMP specific PDE inhibitors have been shown to be promising stimulators for OPC differentiation that boosts CNS repair in MS. However, validating these findings in multiple *in vitro* and *in vivo* models for remyelination is essential before further clinical development.

### Neurons

The lack of myelin in both acute and chronically demyelinated lesions has profound pathophysiological consequences. For instance, Na^+^ channels are redistributed over the demyelinated axolemma as a final compensatory mechanism of neurons in order to maintain nerve conduction although the myelin sheath is lost (149). Progressive axonal and neuronal loss associated with MS eventually causes weakening of neural circuits leading to cognitive and motor impairments (150). Therefore, neuroprotection or repair of neuronal damage may delay, halt, or counter disease progression.

Stimulation of cyclic nucleotide signaling has been shown to increase neuronal resilience by promoting neuroplasticity. Inhibition of PDEs may therefore be an appropriate strategy to induce neuroprotection in MS. Inhibition of specific PDEs is found to enhance neuroplasticity, subsequently increasing neuronal resilience. Vinpocetine, a selective PDE1 inhibitor, can limit oxidative stress, and neuronal damage in a model of vascular dementia (151). PDE2 inhibition was found to improve neuronal plasticity, as observed by an increase in hippocampal long term potentiation (LTP), which is regarded as the underlying physiological correlate of memory (152). The increase in LTP was accompanied by improved object memory performance, both in rats and mice (153). After induction of brain ischemia (154) or in animal models using chronic unpredictable stress (155), the PDE2 inhibitor Bay 60-7550 attenuated the pathological decrease in neuroplasticity related proteins (e.g., BDNF), thereby enhancing neuroplasticity and subsequently neuroprotection. Cilostazol, a PDE3 inhibitor, mediates neuronal repair after induced neuronal loss in the dentate gyrus through an increase in pCREB-mediated hippocampal neural stem cell proliferation (156). The potential of PDE4 inhibition to stimulate neuroplasticity has been studied extensively in the context of learning and memory (37, 157, 158); both non-specific inhibition of the PDE4 gene family (159), as well as targeting of individual PDE4 genes (160), or isoforms (161) are able to increase neuroplasticity and memory functioning. Moreover, neuroprotective and neuroregenerative effects by inhibition of PDE4 have been shown after different types of insults, including SCI (28, 162, 163), striatal neurotoxicity (164–166) and mouse models of Huntington's disease (166, 167). Similarly, PDE5 inhibition by sildenafil and vardenafil can not only improve object memory (168, 169), but also protect against striatal degeneration by stimulation of neuronal surviving pathways, including BDNF and p-CREB expression (170). Interestingly, sildenafil treatment (15 mg/kg administered orally) reduces oxidative stress in mice exposed to noise stress through an increase in free radical scavengers such as super oxide dismutase (SOD) 1, SOD2 and SOD3 (171). Additionally, PDE7 inhibition was found to induce neuroprotective and anti-inflammatory activities in a rat model of Parkinson's disease (172). A reduction in hippocampal apoptosis was also observed after PDE7 inhibition in an Alzheimer mouse model (173). Alzheimer-associated decreases in dendritic spines and plasticity can be counteracted by PDE9 inhibition (174). Finally, PDE10 inhibition increases neuronal survival in a transgenic mouse model of Huntington's disease (175).

As described above, neuroprotective treatment strategies can be achieved by targeting distinct PDE families or isoforms, given the wide applicability of PDE inhibitors to suppress damaging signals such as neuronal apoptosis and oxidative stress, but also to stimulate neuronal survival and repair. Furthermore, inhibition of these PDEs is primarily associated with an improvement in cognitive performance including memory and learning. This latter aspect makes PDE inhibition even more interesting, considering cognitive decline is one of the major symptoms of disease progression in MS (176). However, while inhibition of different types of PDE enzymes has been shown to be beneficial in several models, the exact mechanisms underlying its neuroprotective effects are yet to be elucidated in the context of MS.

## PDE Inhibitors in Clinical Trials as a Therapy for MS

It is clear that PDEs are involved in numerous and different processes in MS. Currently, the majority of MS therapies are focused on reducing disease severity by preventing the infiltration or activation of immune cells in the CNS. However, these therapeutics are unable to halt or reverse disease progression. Therefore, there is an urgent need for the development of new therapeutic strategies. The multimodal effects of PDE inhibitors makes them highly interesting for clinical use to treat MS patients. However, most research regarding PDE inhibitors to date has been performed at the preclinical level and very few clinical trials have been conducted to assess their clinical potential (Table 1).

**Table 1:** Overview of (pre-)clinical studies with PDE inhibitors for the treatment of MS.

**Drug name**	**Indication**	**Route of administration**	**Mode of action**	**Target**	**Status**
AP-1	MS	Oral	Immune modulation	PDE7 and GSK3B	Preclinical
Ibudilast	PPMS; SPMS	Oral; ophthalmic	Anti-inflammatory; neuroprotective	Non-selective PDE inhibitor	Phase II
Revamilast	MS	Oral	Immune modulation	PDE4	inactive
Rolipram	MS	Oral	Facilitates neural transmission; immune modulation	PDE4	Inactive
Sildenafil	RRMS/SPMS	Oral	Increase blood flow	PDE5	Phase II
Small molecules to inhibit PDE7	MS	Oral	Immune modulation	PDE7	Inactive
TDP-101	MS	/	Immune modulation	PDE4B	Preclinical

*MS multiple sclerosis; RRMS relapse remitting MS; PPMS primary progressive MS; SPMS secondary progressive MS; PDE phosphodiesterase; GSK3B glycogen synthase kinase 3 beta GSK3B (GlobalData extraction 25/05/2019)*.

In early 2001, a Phase I/Early Phase II clinical trial was designed to test the dose, tolerability, and efficacy of the PDE4 inhibitor, rolipram as a treatment against CNS inflammation for MS patients (177). In the first stage of the study, six MS patients were enrolled to assess the optimal and safe dose of the compound. Two additional patients with moderate inflammatory brain activity were recruited for the second stage of the study. Even though no difference in clinical disability was observed, rolipram was not well tolerated by the patients. Adverse events such as nausea, vomiting, gastroesophageal reflux, and insomnia were common during the therapy. Moreover, rolipram treatment was accompanied by the unexpected side-effect of an increase in the total amount of contrast enhanced lesions (CEL) per patient when compared to their baseline state demonstrating it has no clinical benefit. As this was the predetermined primary outcome of the study, the trial was terminated in an early phase (177).

Similarly, a double blind, placebo-controlled phase II trial was conducted to evaluate the safety and effects of Ibudilast as a treatment strategy for RRMS patients (178). As discussed above, ibudilast is a non-selective PDE inhibitor that also inhibits the macrophage migration inhibitory factor and toll-like receptor 4 (179, 180). Patients who enrolled in the study received either 30 mg, 60 mg ibudilast, or a placebo every day for 1 year. No difference in lesion activity was observed between the different groups, resulting in an unmet primary endpoint. However, ibudilast treatment seemed to slow brain atrophy, a measure of permanent tissue injury and disease progression in MS (178). Consequently in 2013, the SPRINT-MS phase II clinical trial was established to assess the efficacy and tolerability of ibudilast as a treatment for progressive MS patients (181). Both PPMS and SPMS patients were recruited in the study and received either 50 mg ibudilast or a placebo, twice daily for 96 weeks. The recently published results show that upon ibudilast treatment, the rate of brain atrophy was slowed by 48%. However, as with rolipram, treatment with ibudilast was accompanied by adverse events such as gastrointestinal symptoms, headaches and depression (181).

In 2004, a pilot study was initiated to investigate whether the PDE5 inhibitor, sildenafil citrate, improves cerebral blood perfusion in MS patients (182). MS patients frequently experience a compromised cerebral blood flow which can lead to neuronal cell death. Therefore, it was hypothesized that blood flow perfusion can be increased in these patients through treatment with sildenafil citrate. Both MS patients and healthy volunteers were recruited for this study. MRI scans of the cerebral arteries were taken at baseline prior to treatment, as well as 1 h after sildenafil citrate administration (182). Even though the study was completed within 2 years, the results and outcomes of the trial are yet to be disclosed.

At present, ibudilast is the only PDE inhibitor that has yielded positive results in a clinical setting. The ongoing SPRINT-MS study will validate whether the observed effects of ibudilast on brain atrophy are reproducible, and if it is associated with slower disease progression (181). However, even though ibudilast targets different PDE families, it preferentially targets PDE4 (183). As seen with rolipram, targeting PDE4 in humans is associated with strong adverse effects such as nausea and vomiting, which can compromise the potential use of such inhibitors in clinical settings. There is therefore an urgent need to develop and assess the beneficial effects of PDE isoform inhibitors for their beneficial effects in clinical trials for MS.

## PDE Inhibitors in Other Neurodegenerative Disorders: Relevance for Treating MS

Neuroinflammation and neurodegeneration are central processes involved in a wide range of CNS disorders. Based on the aforementioned cellular effects, it is not surprising that PDE inhibitors have been extensively studied in the context of disorders other than MS. Therefore, multiple lessons can be drawn from studies conducted in other disorders and may be implemented when devising therapeutic applications of PDE inhibitors in MS. Although neuroinflammatory and neurodegenerative processes are identical in many disorders, the underlying pathological causality is highly diverse. Therefore, relevant findings supporting the role of PDE inhibitors in other CNS disorders do not provide conclusive results, but rather show the potential of PDE inhibitors in treating MS. Here we will briefly discuss the potential of PDE inhibitors for treating CNS trauma and Alzheimer's disease (AD), and their relevance for treating MS.

After CNS trauma such as SCI, a chronic neuroinflammatory response occurs that impairs neuroregeneration. PDE4 inhibition has been shown to reduce inflammatory processes in monocytes and lymphocytes (184). Considering the role of these infiltrating immune cells in the pathophysiology of SCI, there have been many studies further investigating the effect of PDE4 inhibition. It was demonstrated that PDE4 inhibition increases axonal regeneration using the PDE4 inhibitor rolipram (144). Moreover, Whitaker et al. found that rolipram protected oligodendrocytes against secondary cell death. Furthermore, it was shown that spinal cord oligodendrocytes express PDE4 A, B, and D, while microglia predominantly express PDE4B (144). Bao et al. have also demonstrated that PDE4 inhibition decreased white matter damage, oxidative stress, and leukocyte infiltration, resulting in cellular protection and locomotor improvements after SCI (184). In addition to PDE4, PDE7 inhibition was also studied in the context of SCI. PDE7 is expressed on both macrophages and neurons (185, 186). Paterniti et al. sought to determine the effect of PDE7 inhibition on secondary processes after SCI. Their data demonstrated that PDE7 reduced spinal cord inflammation, tissue injury, neutrophil infiltration, oxidative stress, and apoptosis after SCI (186). Cognitive impairment is an additional effect of neurodegeneration. PDE4 inhibition can also affect cognitive behavior after trauma. This was demonstrated after traumatic brain injury (TBI), where rolipram rescued the cognitive impairment in rats with TBI, an effect that might be attributable to increased CREB activation during learning (187). The promising results of PDE inhibition in CNS trauma are consistent with the previously described potential of these inhibitors in MS treatments. Reduced neuroinflammatory responses, increased axonal regeneration and decreased oxidative stress levels upon PDE4 inhibition can all halt or prevent disease progression of MS. Furthermore, PDE4 inhibition additionally rescued cognitive impairment in pathological circumstances. Given that 40–65% of MS patients experience cognitive impairments (188), PDE4 inhibition would not only reduce pathological hallmarks in the CNS of MS patients, but would also directly reduce a prominent MS-related symptom.

Neuroinflammation is a major hallmark of AD. The pathological proteins amyloid-beta (Aβ) and tau have also been closely linked to inflammatory responses in the brain (189). In AD, an innate immune response is triggered in the brain when aggregated or misfolded proteins bind to pattern recognition receptors on astrocytes and microglia (190). Subsequent secretion of inflammatory mediators (e.g., TNF-α and IL-1β) aggravate AD pathogenesis and contribute to disease progression. Moreover, neuroinflammation may also be a risk factor or mediator in the onset and/or progression of AD (191). Therefore, modulation of neuroinflammatory and/or neuroprotective processes by general or cell type-specific targeting of PDEs may hold therapeutic potential in the context of treating AD. The effect of PDE modulation on neuroinflammation and neurodegeneration has been studied in multiple *in vitro* and *in vivo* AD models. Inhibition of PDE4 was shown to exhibit anti-inflammatory effects in transgenic AD mice (192, 193). More specific inhibition of the PDE4D isoforms PDE4D4 and PDE4D5 was able to decrease Aβ-induced expression of NFκB, TNFα, and IL-1β (194). In microglia, it was found that Aβ induced increased expression of PDE4B, resulting in higher TNFα release. Inhibition of PDE4B was shown to counter upregulation of TNFα by up to 70% (195). Moreover, ferulic acid, a putative PDE4B2 inhibitor, alleviated increased TNFα and IL-1β levels induced by Aβ (196). Inhibition of the entire PDE4 gene family as well as specific PDE4 genes and isoforms seems to hold anti-inflammatory and neuroprotective potential. Cytotoxicity induced by Aβ was found to be attenuated upon PDE4, PDE5, PDE9, but not PDE3 inhibition in a neuroblastoma cell line (197). Further *in vivo* studies involving intraperitoneal administration of 10 mg/kg PDE5 inhibitor sildenafil to transgenic AD mice resulted in decreased neuroinflammation (122). Protective effects by PDE9 inhibition are supported by the observation that PDE9 inhibition reduced Aβ-induced oxidative stress in transgenic AD mice (198). In addition to anti-inflammatory effects induced by PDE inhibition, functional improvements in memory deficits associated with transgenic AD mouse models have been shown for the inhibition of several PDEs. Chronic treatment with the PDE2 inhibitor BAY60-7550 (0.3 mg/kg for 8 weeks) improved spatial memory in transgenic AD mice (199). Similarly, addition of the PDE3 inhibitor cilostazol to food pellets of Tg-SwDI mice improved cognition as reflected by grooming behavior (200). PDE4 inhibition by rolipram or FFPM restores memory performance in APP/PS1 transgenic mice as well as cognitive impairments induced by streptozotocin or natural aging (193, 201, 202). Specific inhibition of PDE4D (daily subcutaneous injections with GEBR-7b (0.001 mg/kg) for 3 weeks) improved spatial memory in APPswe/PS1dE9 mice (203). In APP/PS1 mice, memory improvements were found after PDE5 inhibition using administration of sildenafil or icariin (122, 204, 205). Daily treatment with the PDE7 inhibitor S14 (5 mg/kg, intraperitoneally) for 4 weeks improved memory performance in APP/PS1 mice (173). PDE9 inhibition using the inhibitor BAY-73-6691 improved memory deficits in tg2576 mice and mice subjected to intracerebroventricular injection of Aβ25-35 (174, 198). Additionally, the combination of subefficacious doses of a PDE4 (roflumilast) and PDE5 (vardenafil) inhibitor could improve memory in transgenic APPswe mice (206). These preclinical findings indicate that neuroinflammatory and memory-associated processes can be modulated through inhibition of different PDE gene families. As both the signaling cascades involving cAMP and cGMP seem to be affected in AD patients (207–210), inhibition of cAMP- and cGMP-specific PDEs holds potential as therapeutic strategy. However, clinical studies investigating the memory-enhancing potential of PDE inhibitors show inconsistent effects (157, 211). Several changes in PDE expression have been observed in AD brains which seem to be dependent on brain region, cell-type and disease progression (33). Since neuroinflammatory processes may partially mediate initiation or progression of AD, PDE inhibition may provide an exploitable strategy to interfere with these processes. Both in AD and MS, PDE4B(2) has shown to be critically involved in mediating the pro-inflammatory responses of phagocytes in the CNS. Also, inhibition of specific PDE4D isoforms has been shown to possess anti-inflammatory properties in AD, demonstrating the potential of these inhibitors to be explored in the pathogenesis of MS. Apart from direct effects on neuroinflammation, inhibition of PDEs may decrease neuroinflammation and neurodegeneration by influencing AD pathology and neuroplasticity. However, these indirect effects are beyond the scope of this review. Therefore, it would be best if future studies will indicate the expression regulation and role of PDEs per cell type in order to more specifically target cellular processes underlying neuroinflammation and neurodegeneration in both AD and MS.

## Concluding Remarks

The role of PDE inhibitors to modulate neuroinflammatory and neuroreparative processes has gained tremendous interest over the last several years. It is becoming clear that targeting of PDE families can modify multiple cellular key players involved in a variety of processes involved in MS pathogenesis. Due to this multifactorial effect, inhibiting a single PDE family is often accompanied with severe side effects, hampering their translation for a clinical application. Nevertheless, different PDE families are shown to be beneficial in different phases of MS. For example, in the initial phase of MS when BBB integrity is lost, not cGMP but rather cAMP-specific PDE inhibitors are considered a viable therapeutic strategy. Elevating cAMP levels in endothelial cells increased the expression of tight junctions, while elevating cAMP levels in astrocytes decreased the expression of adhesion molecules, subsequently creating a synergistic effect that prevents peripheral lymphocyte infiltration into the CNS via the BBB. During RRMS, there is already an ongoing active pro-inflammatory response. Therefore, disease progression may be halted through the use of cAMP-specific PDE inhibitors to either directly modulate Th1 and Th17 responses or to increase Treg populations to regulate immune homeostasis. Elevating cAMP levels in phagocytes diminishes the secretion of pro-inflammatory cytokines and subsequently lowers the pro-inflammatory phenotype of these cells. Before CNS repair can be initiated in MS patients, myelin debris needs to be internalized at the lesion site by these phagocytes. However, the phagocytic properties of these cells are not stimulated by the increase of intracellular cAMP levels, but rather by the increase of cGMP levels specifically. Therefore, cGMP-specific PDE inhibitors are considered a potential therapeutic strategy for promoting repair processes in later stages of MS. In the context of OPC differentiation, both cAMP and cGMP specific PDE inhibitors have shown their potential. However, these findings require replication be validated and the potential of the inhibitors should be explored further in the context of progressive MS. Finally, enhancing neuroplasticity is considered a possible strategy for promoting functional recovery in MS patients. Multiple PDE inhibitors have been shown to be neuroprotective and to enhance neuroplasticity *in vitro* and *in vivo*, however their efficacy in the context of MS remains unexplored.

Although promising results were obtained in pre-clinical studies, contradictory results were observed in PDE KO animals compared to pharmacological inhibition. However, directly comparing these findings is difficult given the developmental differences in these animals due to the permanent absence of the PDE enzyme throughout the animal's life. Compensatory mechanism are potentially being activated early in the life of PDE KO animals causing an increased expression of other PDE families, genes or isoforms. The development of conditional KO animals can therefore lead to new promising results to confirm the involvement of specific PDEs in pathological conditions. Furthermore, clinical studies using PDE inhibitors often show severe side effects due to the multifactorial effects of PDE inhibitors on multiple cellular processes. PDE isoforms show specific cellular compartmentalization, creating distinct signalosomes within different cells. Therefore, identifying which PDE genes and isoforms underlie distinct pathogenic processes in MS can create a more targeted approach for modifying specific key players during different phases of MS. As such, targeting specific PDE isoforms can further lower the occurrence of adverse events. Taken together, identifying the key PDE families, genes and isoforms involved in specific phases and processes may lead to the development of a tailor-made approach for treating MS.

## Author Contributions

All authors listed have made a substantial, direct and intellectual contribution to the work, and approved it for publication.

### Conflict of Interest Statement

TV and JP have a proprietary interest in selective PDE4D inhibitors for the treatment of MS. The remaining authors declare that the research was conducted in the absence of any commercial or financial relationships that could be construed as a potential conflict of interest.
